# Crystal structure and Hirshfeld surface analysis of 4-oxo-3-phenyl-2-sulfanyl­idene-5-(thio­phen-2-yl)-3,4,7,8,9,10-hexa­hydro-2*H*-pyrido[1,6-*a*:2,3-*d*′]di­pyrimidine-6-carbo­nitrile

**DOI:** 10.1107/S2056989024001658

**Published:** 2024-02-20

**Authors:** Farid N. Naghiyev, Victor N. Khrustalev, Mehmet Akkurt, Huseyn M. Mamedov, Ajaya Bhattarai, Ali N. Khalilov, İbrahim G. Mamedov

**Affiliations:** a Department of Chemistry, Baku State University, Z. Khalilov str. 23, Az, 1148, Baku, Azerbaijan; b Peoples’ Friendship University of Russia (RUDN University), Miklukho-Maklay St. 6, Moscow 117198, Russian Federation; c N. D. Zelinsky Institute of Organic Chemistry RAS, Leninsky Prosp. 47, Moscow, 119991, Russian Federation; d Department of Physics, Faculty of Sciences, Erciyes University, 38039 Kayseri, Türkiye; e Faculty of Physics, Baku State University, Z. Khalilov str. 23, Az, 1148 Baku, Azerbaijan; f Department of Chemistry, M.M.A.M.C (Tribhuvan University) Biratnagar, Nepal; g "Composite Materials" Scientific Research Center, Azerbaijan State Economic University (UNEC), H. Aliyev str. 135, Az 1063, Baku, Azerbaijan; University of Neuchâtel, Switzerland

**Keywords:** crystal structure, hydrogen bonds, heterocycle, Hirshfeld surface analysis

## Abstract

In the title compound, C_21_H_15_N_5_OS_2_, mol­ecular pairs are linked by N—H⋯N hydrogen bonds along the *c-*axis direction and C—H⋯S and C—H⋯O hydrogen bonds along the *b-*axis direction, with *R* ^2^_2_(12) and *R* ^2^_2_(16) motifs, respectively, thus forming layers parallel to the (10  ) plane. In addition, C=S⋯π and C≡N⋯π inter­actions between the layers ensure crystal cohesion. The Hirshfeld surface analysis indicates that the major contributions to the crystal packing are H⋯H (43.0%), C⋯H/H⋯C (16.9%), N⋯H/H⋯N (11.3%) and S⋯H/H⋯S (10.9%) inter­actions.

## Abstract 2

In the title compound, mol­ecular pairs are linked by N—H⋯N hydrogen bonds along the *c-*axis direction and C—H⋯S and C—H⋯O hydrogen bonds along the *b*-axis direction, with 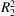 (12) and 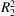 (16) motifs, respectively, thus forming layers parallel to the (10 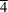 ) plane. In addition, C=S⋯π and C≡N⋯π inter­actions between the layers ensure crystal cohesion.

## 1. Chemical context

Heterocyclic systems are an important group of organic compounds. Synthetic chemistry has grown abundantly over the past few decades and recently developed heterocyclic systems have found diverse research and commercial applications, especially in the pharmaceutical and chemical industries (Maharramov *et al.*, 2021 ▸, 2022 ▸; Erenler *et al.*, 2022 ▸; Akkurt *et al.*, 2023 ▸). These compounds have also found wide implementations in diverse fields of chemical science, including in coordination chemistry (Gurbanov *et al.*, 2021 ▸; Mahmoudi *et al.*, 2021 ▸), medicinal chemistry (Dönmez & Türkyılmaz, 2022 ▸; Askerova, 2022 ▸) and materials science (Velásquez *et al.*, 2019 ▸; Afkhami *et al.*, 2019 ▸). Pyridodi­pyrimidines are a specific group of heterocyclic systems that contain a fused tricyclic system with four or five nitro­gen atoms in their structure. These compounds are analogues of tetra- or penta-aza-anthracene or phenanthrene and usually exist in either a linear or an angular form. This moiety is present in drugs, and in recent years it has been studied in the development of new active compounds, as evidenced by numerous publications (Yousif *et al.*, 2021 ▸; Sobhi & Faisal, 2023 ▸). Derivatives comprising the pyridodi­pyrimidine skeleton show diverse biological activities, such as anti­tumour activity, inhibiting di­hydro­folate reductases or tyrosine kinases, anti-inflammatory activity, anti­hypertensive activity, anti­bacterial activity, anti­convulsant activity, calcium channel antagonist activity, *etc*. Historical and modern synthetic approaches for the preparation of these systems have been reviewed recently (Atalay *et al.*, 2022 ▸; Hammouda *et al.*, 2023 ▸). Thus, in the framework of our studies in heterocyclic chemistry (Naghiyev *et al.*, 2020 ▸, 2021 ▸, 2022 ▸; Khalilov *et al.*, 2022 ▸), we report the synthesis and characterization of the title compound, 4-oxo-3-phenyl-2-sulfanyl­idene-5-(thio­phen-2-yl)-3,4,7,8,9,10-hexa­hydro-2*H*-pyrido[1,6-*a*:2,3-*d*′]di­pyrimidine-6-carbo­nitrile. 
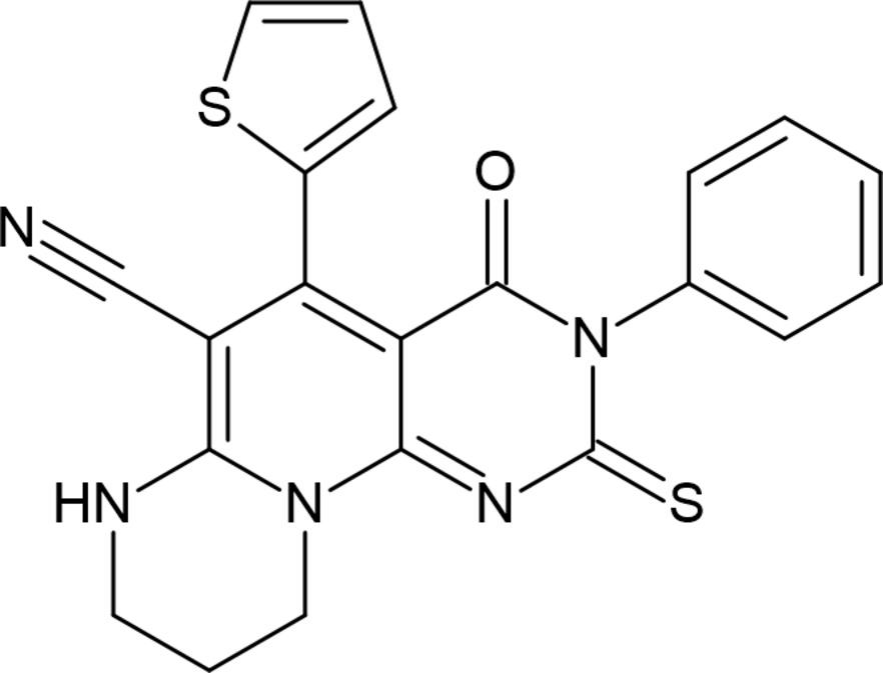


## 2. Structural commentary

**Figure 1:**
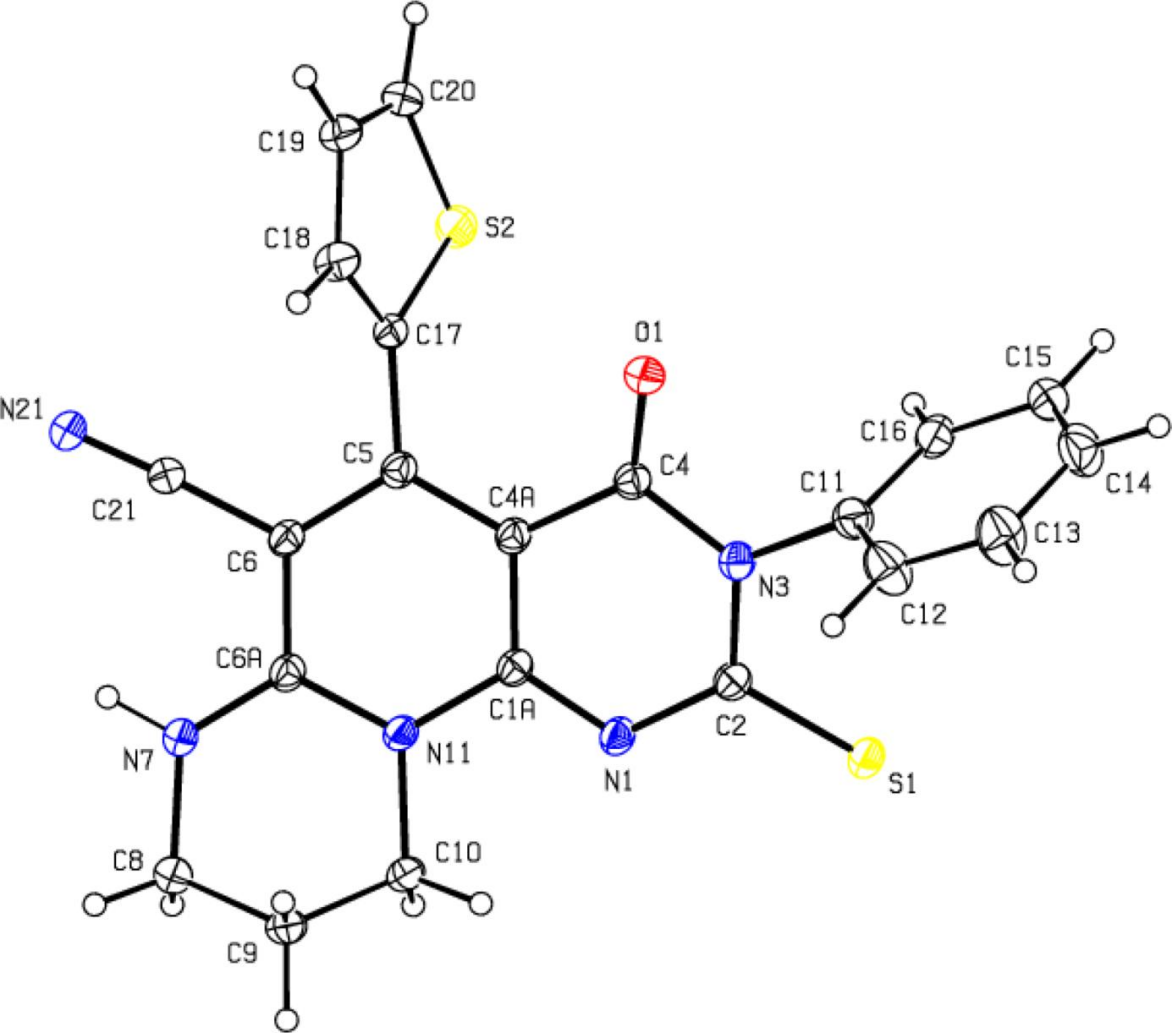
The mol­ecular structure, showing the atom labelling and displacement ellipsoids drawn at the 30% probability level. Only the major component of the disorder is shown.

The thio­phene ring (S2/C17–C20; Fig. 1 ▸) in the title compound is disordered over two sites in a 0.787 (3):0.213 (3) ratio by an approximate rotation of 180° about the C5—C17 bond. The phenyl ring (C11-C16) is also disordered over two positions with the same ratio. In the 1,3-diazinane ring (N7/N11/C6*A*/C8–C10), the middle carbon atom (C9) is similarly disordered. The ten-membered 2,3,4,8-tetra­hydro­pyrido[2,3-*d*]pyrimidine ring system (N1/N3/N11/C1*A*/C2/C4/C4*A*/C5/C6/C6*A*) has a nearly planar conformation (r.m.s. deviation = 0.1183 Å). The dihedral angles between the major and minor components of the disordered phenyl (C11–C16 and C11/C12–C16*A*) and thio­phene (S2/C17–C20 and S2*A*/C17/C18*A*–C20*A*) rings are 20.3 (9) and 6.7 (7)°, respectively, and these disordered components make dihedral angles of 71.9 (3), 88.0 (4)° and 64.0 (2), 70.6 (4)°, respectively, with the ten-membered ring system. The geometric parameters are normal and comparable to those of related compounds described in the *Database survey* section.

## 3. Supra­molecular features and Hirshfeld surface analysis

**Figure 2:**
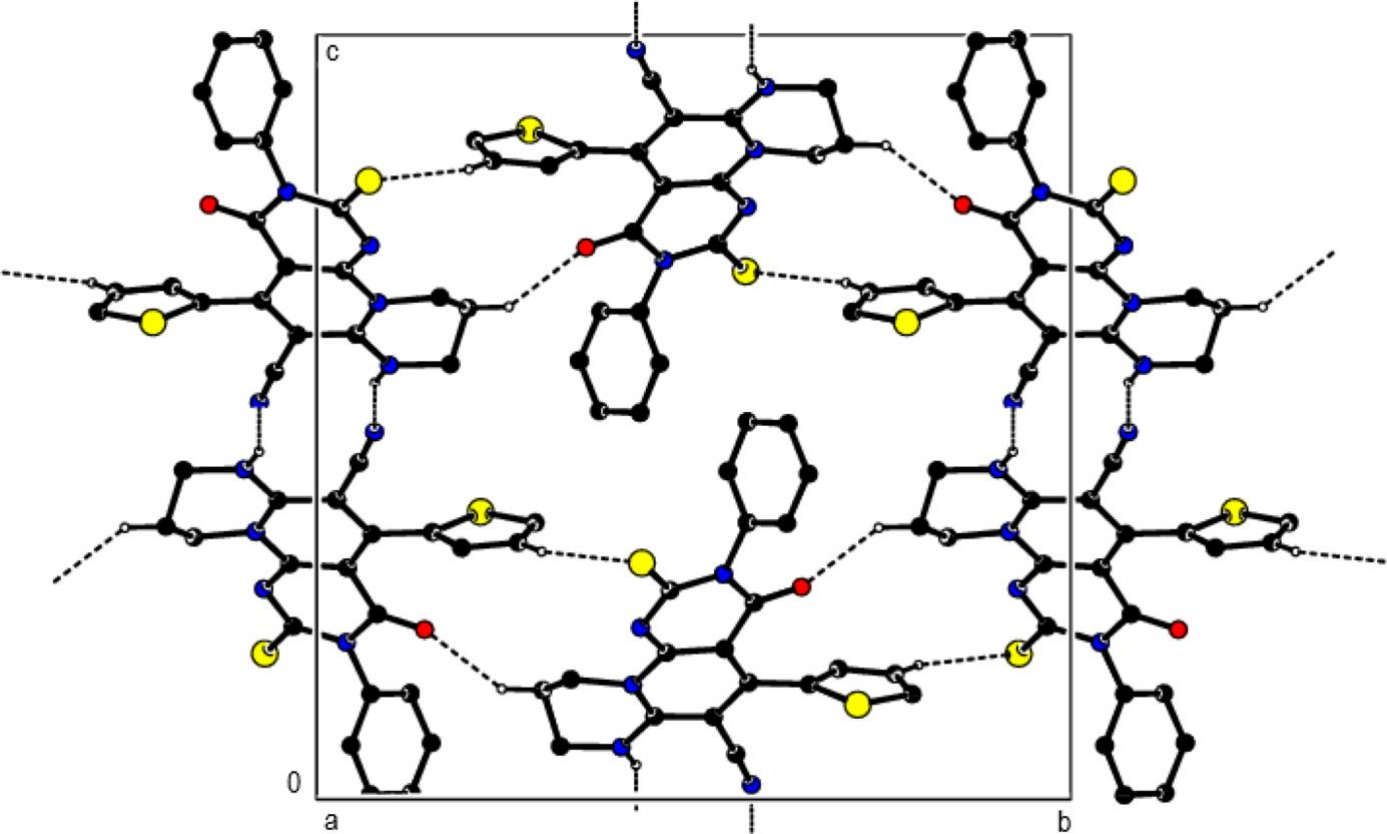
View of the N—H⋯N, C—H⋯O and C—H⋯S hydrogen bonds down the *a*-axis. Only the major component of the disorder and the H atoms involved are shown.

**Figure 3:**
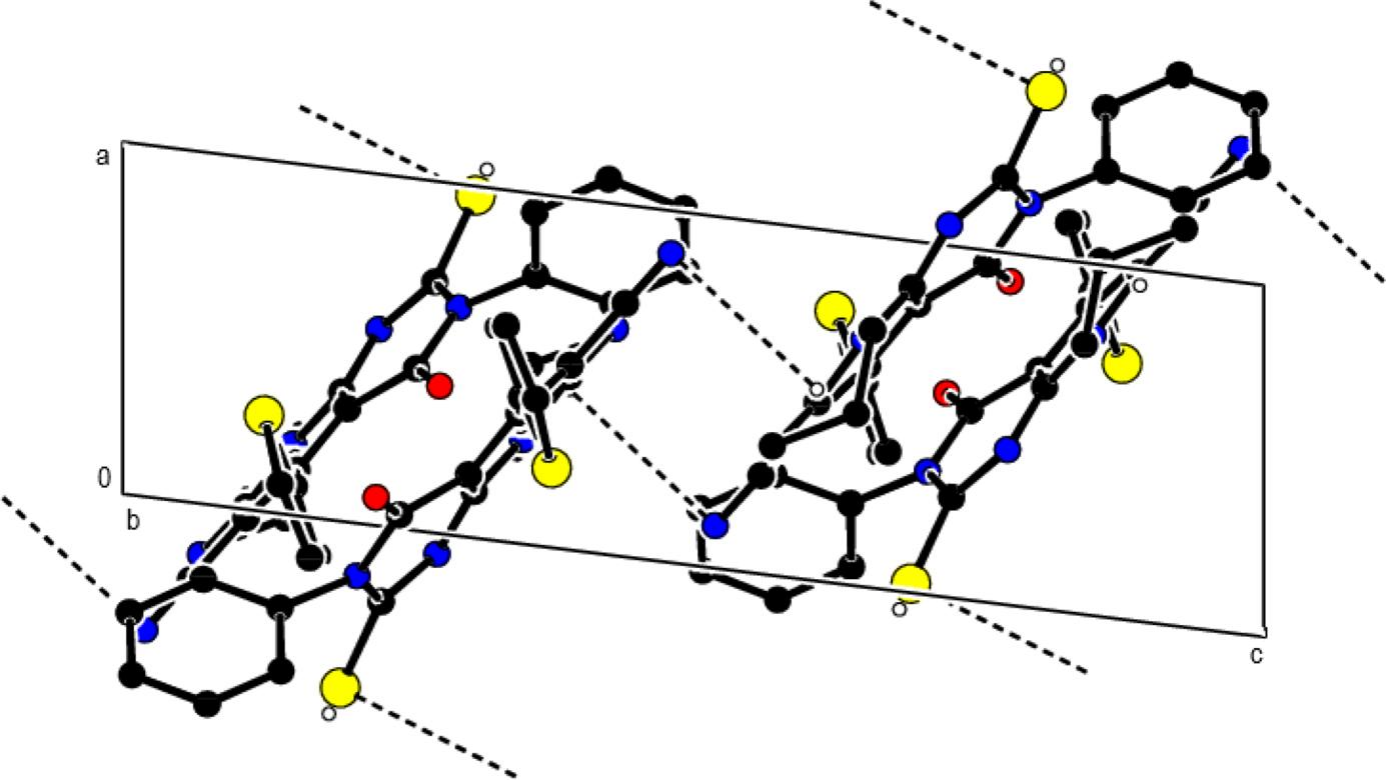
View of the π–π and C—N⋯π and C—S⋯π inter­actions down the *b*-axis. Only the major component of the disorder is shown. All H atoms are omitted for clarity.

In the crystal, mol­ecular pairs are linked by N—H⋯N hydrogen bonds along the *c*-axis direction and C—H⋯S and C—H⋯O hydrogen bonds along the *b*-axis direction, with 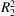 (12) and 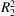 (16) motifs, respectively (Bernstein *et al.*, 1995 ▸; Table 1 ▸; Fig. 2 ▸). They form layers parallel to the (10 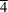 ) plane. Crystal cohesion between the layers is ensured by C=S⋯π and C≡N⋯π inter­actions [(C2)S1⋯*Cg*6^a^ = 3.4304 (9) Å, C2(S1)⋯*Cg*6^a^ = 3.643 (2) Å, C2=S1⋯*Cg*6^a^ = 83.57 (8)°; (C21)N21⋯*Cg*5^b^ = 3.330 (4) Å, C21(N21)⋯*Cg*5^b^ = 3.613 (4) Å, C21≡N21⋯*Cg*5^b^ = 94.91 (15)°; symmetry codes: (a) −1 + *x*, *y*, *z*; (b) 2 − *x*, 1 − *y*, 2 − *z*; *Cg*5 and *Cg*6 are the centroids of the N7/N11/C6*A*/C8/C9*A*/C10 and N11/C1*A*/C4*A*/C5/C6/C6*A* rings] (Table 1 ▸; Fig. 3 ▸).

**Figure 4:**
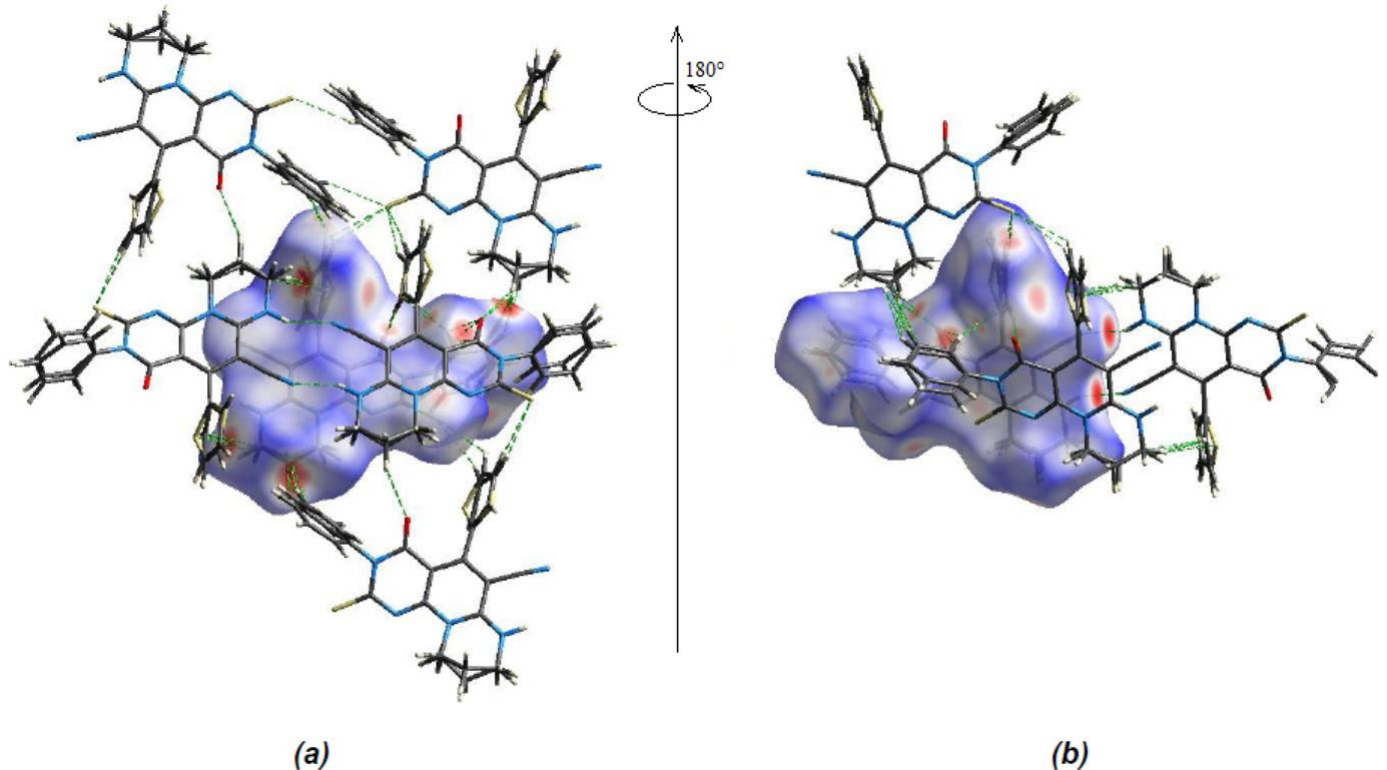
(*a*) Front and (*b*) back sides of the three-dimensional Hirshfeld surface of the compound mapped over *d*_norm_.

**Figure 5:**
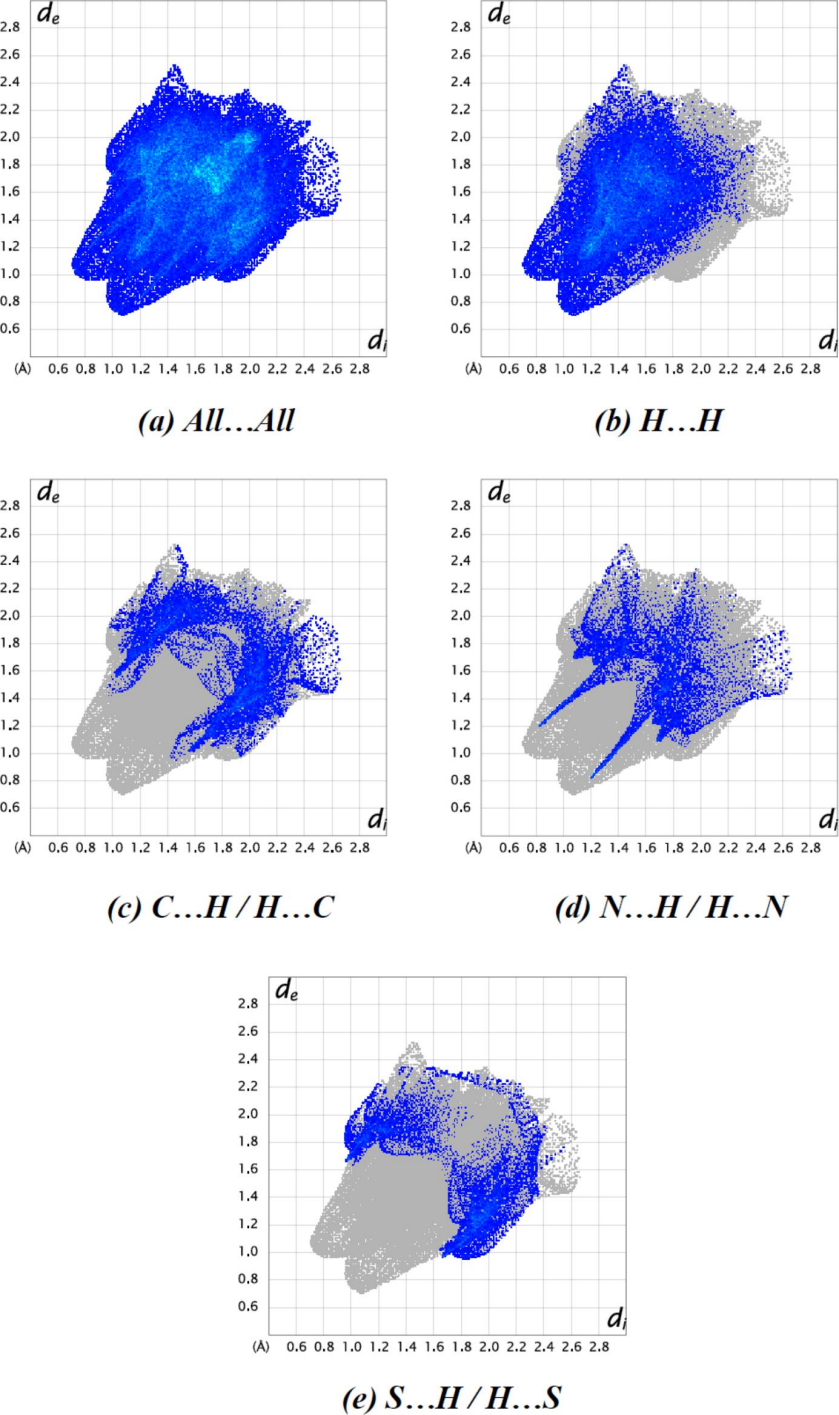
The two-dimensional fingerprint plots of the title compound, showing (*a*) all inter­actions, and delineated into (*b*) H⋯H, (*c*) C⋯H/H⋯C, (*d*) N⋯H/H⋯N and (*e*) S⋯H/H⋯S inter­actions. [*d*_e_ and *d*_i_ represent the distances from a point on the Hirshfeld surface to the nearest atoms outside (external) and inside (inter­nal) the surface, respectively].

Two-dimensional fingerprint plots and Hirshfeld surfaces were produced using *Crystal Explorer 17.5* (Spackman *et al.*, 2021 ▸) to qu­antify the inter­molecular inter­actions. The *d*_norm_ surfaces are mapped over a fixed colour scale from −0.4663 (red) to +1.2045 (blue) a.u. Red spots on the surface corres­pond to N—H⋯N, C—H⋯O and C—H⋯S inter­actions (Tables 1 ▸ and 2 ▸; Fig. 4 ▸ *a*,*b*). The most significant inter­atomic contact is H⋯H, because it contributes the most to the crystal packing (43.0%, Fig. 5 ▸ *b*). Other significant contributions are from C⋯H/H⋯C (16.9%, Fig. 5 ▸ *c*), N⋯H/H⋯N (11.3%, Fig. 5 ▸ *d*) and S⋯H/H⋯S (10.9%, Fig. 5 ▸ *e*) inter­actions. The following inter­actions have minor contributions: O⋯H/H⋯O (7.2%), C⋯C (3.4%), N⋯C/C⋯N (3.1%), S⋯C/C⋯S (2.0%), N⋯N (1.3%) and S⋯N/N⋯S (0.8%).

## 4. Database survey

A search of the Cambridge Structural Database (CSD, Version 5.43, last update November 2022; Groom *et al.*, 2016 ▸) for the central ten-membered ring *2,3,4,8-tetra­hydro­pyrido[2,3-d]pyrimidine* yielded four hits, *viz*. 11-(amino­methyl­idene)-8,9,10,11-tetra­hydro­pyrido[2′,3′:4,5]pyrimido[1,2-*a*]aze­pin-5(7*H*)-one (CSD refcode HECLUZ; Khodjaniyazov *et al.*, 2017 ▸), 7-amino-1,3-dimethyl-5-(4-nitro­phen­yl)-2,4-dioxo-1,2,3,4-tetra­hydro­pyrido(2,3-*d*)pyrimidine-6-carbo­nitrile (NIFBUA; Zhou *et al.*, 2007 ▸), 3-(4-fluoro­phen­yl)-1,5,7-tri­methyl-1,2,3,4-tetra­hydro­pyrido(2,3-*d*)pyrimidine-2,4-dione (Patel *et al.*, 2007 ▸) and 2-(4-chloro-3-methyl­phen­oxy)-3-(4-chloro­phen­yl)-5-methyl-8,9,10,11-tetra­hydro-1-benzothieno(2′,3′:2,3)pyrido(4,5-*d*)pyrimidin-4(3*H*)-one di­chloro­methane solvate (JAYKOK; Liu *et al.*, 2005 ▸). In HECLUZ, hydrogen bonds with a 16-membered ring and three chain motifs are generated by N—H⋯N and N—H⋯O contacts. The amino group is located close to the nitro­gen atoms N1 and N8 of an inversion-related mol­ecule, forming hydrogen bonds with 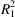 (4) and 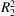 (12) graph-set motifs. This amino group also forms a hydrogen bond with the C=O oxygen atom of a mol­ecule translated along the *a-*axis direction, which links the mol­ecules into 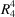 (16) rings. Hydrogen-bonded chains are formed along [100] by alternating 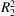 (12) and 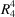 (16) rings. These chains are stabilized by inter­molecular π–π stacking inter­actions between the pyridine and pyrimidine rings [centroid–centroid distance = 3.669 (2) Å; symmetry operation 1 − *x*, 1 − *y*, 1 − *z*]. In NIFBUA, mol­ecules are linked by N—H⋯O, C—H⋯O and C—H⋯N hydrogen bonds, forming a three-dimensional network. In HIFREU, a diverse set of weak inter­molecular C—H⋯π, π–π and C—H⋯O inter­actions link the mol­ecules into sheets. The C—H⋯O inter­actions generate centrosymmetric rings with an 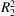 (14) graph-set motif and chains with a *C*(8) motif. In JAYKOK, the mol­ecules are connected in the form of zigzag ribbons along the *b*-axis direction by C—H⋯π and C—Cl⋯π inter­actions. van der Waals inter­actions between the ribbons ensure the cohesion of the crystal structure.

## 5. Synthesis and crystallization

A solution of 6-amino-9-iso­cyano-8-(thio­phen-2-yl)-3,4-di­hydro-2*H*-pyrido[1,2-*a*]pyrimidine-7-carbo­nitrile (3.5 mmol) and potassium hydroxide (3.5 mmol) was stirred in DMF (25 mL) for 2 h at room temperature. Phenyl iso­thio­cyanate (3.5 mmol) was added dropwise to the reaction mixture and it was stirred for 2 h. The reaction mixture was kept for 48 h at room temperature and acidified with 5 mL (37% HCl) solution. The precipitate was filtered and recrystallized from an ethanol water (3:1 ratio) solution. The title compound was obtained in 77% yield, m.p. 469–470 K.

^1^H NMR (300 MHz, DMSO-*d*_6_, ppm.): 1.95 (*m*, 2H, CH_2_); 3.59 (*t*, 2H, CH_2_); 4.06 (*t*, 2H, CH_2_); 7.31–7.51 (*m*, 6H, 5CH_arom._ + 1H, thioph.); 7.54 (*d*, 1H, thioph.); 7.89 (*d*, 1H, thioph.); 8.40 (*s*, 1H, NH). ^13^C NMR (75 MHz, DMSO-*d*_6_, ppm): 19.84 (CH_2_), 41.22 (CH_2_), 43.68 (CH_2_), 53.58 (=C_tert._), 98.75 (=C_tert._), 119.67 (CN), 122.94 (2CH_arom._), 126.28 (CH_arom._), 126.91 (C_thioph._), 128.43 (CH_thioph._), 129.29 (CH_thioph._), 131.64 (CH_thioph._), 132.72 (2CH_arom._), 135.97 (C_arom._), 147.11 (=C_tert._), 149.45 (=C_tert._), 152.32 (N—C=O), 161.60 (=C_tert._), 179.85 (N—C=S).

## 6. Refinement

Crystal data, data collection and structure refinement details are summarized in Table 3 ▸. The thio­phene ring (S2/C17–C20) is disordered over two sites related by an approximate rotation of 180° about the C5—C17 bond in a 0.787 (3):0.213 (3) ratio. The phenyl ring (C11–C16) is also disordered over two sites in a 0.787 (3):0.213 (3) ratio. The minor occupancy component of the phenyl ring was restrained to be planar, using FLAT commands. The middle carbon atom (C9) in the 1,3-diazinane ring (N7/N11/C6*A*/C8–C10) is similarly disordered. EADP in *SHELXL* was used for the *U*_ij_ values of equivalent atom pairs (*e.g*., S2 and *S2*A**) and SADI was employed for the disordered components to restrain the bond lengths and angles of the major and minor components to be the same within an e.s.d. of 0.02 Å, to ensure chemically reasonable bond length and angle values. The C-bound H atoms were placed in calculated positions (0.95–0.99 Å) and refined as riding atoms with *U*_iso_(H) = 1.2*U*_eq_(C). The N-bound H atoms were located in a difference map and freely refined.

## Supplementary Material

Crystal structure: contains datablock(s) I. DOI: 10.1107/S2056989024001658/tx2082sup1.cif

Structure factors: contains datablock(s) I. DOI: 10.1107/S2056989024001658/tx2082Isup2.hkl

Supporting information file. DOI: 10.1107/S2056989024001658/tx2082Isup3.cml

CCDC reference: 2333770

Additional supporting information:  crystallographic information; 3D view; checkCIF report

## Figures and Tables

**Table 1:** Hydrogen-bond geometry (Å, °)

*D*—H⋯*A*	*D*—H	H⋯*A*	*D*⋯*A*	*D*—H⋯*A*
N7—H7⋯N21^i^	0.89 (2)	2.14 (2)	2.976 (3)	157 (2)
C9—H9*B*⋯O1^ii^	0.99	2.34	3.197 (3)	144
C16—H16⋯S2*A* ^iii^	0.95	2.73	3.58 (2)	149
C19—H19⋯S1^iv^	0.95	2.76	3.652 (5)	156

Symmetry codes: (i) 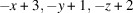 ; (ii) 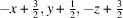 ; (iii) 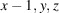 ; (iv) 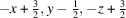 .

**Table 2:** Summary of short inter­atomic contacts (Å). Atoms belonging to the minor disorder components are indicated by an asterisk (*).

*H16*A*⋯O1	2.34	−1 + *x*, *y*, *z*
*H9*B*⋯O1	2.34	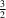 − *x*, 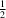 + *y*, 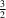 − *z*
*H9*C*⋯*H15	1.87	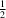 − *x*, 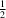 + *y*, 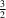 − *z*
*H13⋯S1	2.93	−*x*, 1 − *y*, 1 − *z*
*H18*A*⋯*H8*D*	1.87	2 − *x*, 1 − *y*, 2 − *z*
H7⋯N21	2.14	3 − *x*, 1 − *y*, 2 − *z*
*H13*A*⋯*H20	2.26	− 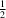 + *x*, 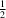 − *y*, − 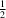 + *z*
*H20*A*⋯*H14	2.58	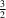 + *x*, 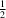 − *y*, 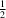 + *z*
*H13*A*⋯*H12	2.46	1 − *x*, 1 − *y*, 1 − *z*

**Table 3:** Experimental details

Crystal data	
Chemical formula	C_21_H_15_N_5_OS_2_
*M*_r_	417.50
Crystal system, space group	Monoclinic, *P*2_1_/*n*
Temperature (K)	100
*a*, *b*, *c* (Å)	5.63465 (3), 18.02763 (13), 18.40115 (12)
β (°)	97.1649 (6)
*V* (Å^3^)	1854.58 (2)
*Z*	4
Radiation type	Cu *K*α
μ (mm^−1^)	2.81
Crystal size (mm)	0.31 × 0.05 × 0.05

Data collection	
Diffractometer	XtaLAB Synergy, Dualflex, HyPix
Absorption correction	Gaussian (*CrysAlis PRO*; Rigaku OD, 2022 ▸)
*T*_min_, *T*_max_	0.495, 1.000
No. of measured, independent and observed [*I* > 2σ(*I*)] reflections	39427, 3946, 3842
*R*_int_	0.032
(sin θ/λ)_max_ (Å^−1^)	0.634

Refinement	
*R*[*F* ^2^ > 2σ(*F* ^2^)], *wR*(*F* ^2^), *S*	0.047, 0.130, 1.09
No. of reflections	3946
No. of parameters	299
No. of restraints	16
H-atom treatment	H atoms treated by a mixture of independent and constrained refinement
Δρ_max_, Δρ_min_ (e Å^−3^)	0.46, −0.35

Computer programs: *CrysAlis PRO* (Rigaku OD, 2022 ▸), *SHELXT* (Sheldrick, 2015*a* ▸), *SHELXL* (Sheldrick, 2015*b* ▸), *ORTEP-3 for Windows* (Farrugia, 2012 ▸) and *PLATON* (Spek, 2020 ▸).

## supplementary crystallographic information

### Crystal data

**Table:**

C_21_H_15_N_5_OS_2_	*F*(000) = 864
*M**_r_* = 417.50	*D*_x_ = 1.495 Mg m^−^^3^
Monoclinic, *P*2_1_/*n*	Cu *K*α radiation, λ = 1.54184 Å
*a* = 5.63465 (3) Å	Cell parameters from 25424 reflections
*b* = 18.02763 (13) Å	θ = 2.4–77.5°
*c* = 18.40115 (12) Å	µ = 2.81 mm^−^^1^
β = 97.1649 (6)°	*T* = 100 K
*V* = 1854.58 (2) Å^3^	Needle, orange
*Z* = 4	0.31 × 0.05 × 0.05 mm

### Data collection

**Table:**

XtaLAB Synergy, Dualflex, HyPix diffractometer	3842 reflections with *I* > 2σ(*I*)
Radiation source: micro-focus sealed X-ray tube	*R*_int_ = 0.032
φ and ω scans	θ_max_ = 77.8°, θ_min_ = 3.5°
Absorption correction: gaussian (CrysAlisPro; Rigaku OD, 2022)	*h* = −7→6
*T*_min_ = 0.495, *T*_max_ = 1.000	*k* = −22→22
39427 measured reflections	*l* = −23→23
3946 independent reflections	

### Refinement

**Table:**

Refinement on *F*^2^	Primary atom site location: difference Fourier map
Least-squares matrix: full	Secondary atom site location: difference Fourier map
*R*[*F*^2^ > 2σ(*F*^2^)] = 0.047	Hydrogen site location: mixed
*wR*(*F*^2^) = 0.130	H atoms treated by a mixture of independent and constrained refinement
*S* = 1.09	*w* = 1/[σ^2^(*F*_o_^2^) + (0.0624*P*)^2^ + 1.7979*P*] where *P* = (*F*_o_^2^ + 2*F*_c_^2^)/3
3946 reflections	(Δ/σ)_max_ = 0.003
299 parameters	Δρ_max_ = 0.46 e Å^−^^3^
16 restraints	Δρ_min_ = −0.34 e Å^−^^3^

### Special details

**Table:**

Geometry. All esds (except the esd in the dihedral angle between two l.s. planes) are estimated using the full covariance matrix. The cell esds are taken into account individually in the estimation of esds in distances, angles and torsion angles; correlations between esds in cell parameters are only used when they are defined by crystal symmetry. An approximate (isotropic) treatment of cell esds is used for estimating esds involving l.s. planes.

### Fractional atomic coordinates and isotropic or equivalent isotropic displacement parameters (Å^2^)

**Table:**

	*x*	*y*	*z*	*U*_iso_*/*U*_eq_	Occ. (<1)
S1	0.02652 (9)	0.56892 (3)	0.69073 (3)	0.03115 (15)	
S2	0.72669 (14)	0.28256 (5)	0.87597 (5)	0.0385 (3)	0.787 (3)
S2A	1.1539 (16)	0.3143 (6)	0.8247 (7)	0.0342 (13)	0.213 (3)
O1	0.5802 (3)	0.35719 (8)	0.72211 (8)	0.0330 (3)	
N1	0.4413 (3)	0.57036 (9)	0.77539 (9)	0.0271 (4)	
C1A	0.6322 (3)	0.53620 (11)	0.80803 (10)	0.0254 (4)	
C2	0.2865 (4)	0.53163 (11)	0.72644 (11)	0.0270 (4)	
N3	0.3507 (3)	0.46068 (10)	0.70538 (9)	0.0281 (4)	
C4	0.5389 (4)	0.41982 (11)	0.74252 (11)	0.0262 (4)	
C4A	0.6778 (3)	0.45923 (11)	0.80310 (10)	0.0251 (4)	
C5	0.8706 (3)	0.42724 (11)	0.84720 (11)	0.0255 (4)	
C6	1.0250 (3)	0.47366 (11)	0.89208 (11)	0.0266 (4)	
C6A	0.9921 (4)	0.55219 (11)	0.89144 (10)	0.0263 (4)	
N7	1.1448 (3)	0.59618 (10)	0.93177 (10)	0.0320 (4)	
H7	1.278 (3)	0.5768 (15)	0.9549 (15)	0.048 (8)*	
C8	1.1322 (4)	0.67714 (12)	0.93061 (14)	0.0378 (5)	
H8A	1.2951	0.6985	0.9390	0.045*	0.915 (5)
H8B	1.0405	0.6951	0.9697	0.045*	0.915 (5)
H8C	1.2530	0.6960	0.9005	0.045*	0.085 (5)
H8D	1.1768	0.6956	0.9812	0.045*	0.085 (5)
C9	1.0108 (5)	0.70013 (13)	0.85711 (13)	0.0335 (6)	0.915 (5)
H9A	1.1097	0.6855	0.8186	0.040*	0.915 (5)
H9B	0.9914	0.7547	0.8555	0.040*	0.915 (5)
C9A	0.899 (3)	0.7088 (11)	0.9022 (12)	0.0335 (6)	0.085 (5)
H9C	0.9231	0.7592	0.8831	0.040*	0.085 (5)
H9D	0.8002	0.7134	0.9429	0.040*	0.085 (5)
C10	0.7676 (4)	0.66313 (11)	0.84300 (12)	0.0326 (5)	
H10A	0.6634	0.6814	0.8786	0.039*	0.915 (5)
H10B	0.6911	0.6756	0.7931	0.039*	0.915 (5)
H10C	0.8202	0.6784	0.7959	0.039*	0.085 (5)
H10D	0.5951	0.6749	0.8404	0.039*	0.085 (5)
N11	0.7967 (3)	0.58104 (9)	0.85025 (9)	0.0263 (4)	
C11	0.2322 (4)	0.42875 (12)	0.63790 (12)	0.0327 (5)	
C12	0.2858 (14)	0.4548 (5)	0.5706 (3)	0.0463 (17)	0.596 (9)
H12	0.4075	0.4911	0.5692	0.056*	0.596 (9)
C13	0.1650 (13)	0.4288 (4)	0.5059 (3)	0.0523 (15)	0.596 (9)
H13	0.2012	0.4474	0.4603	0.063*	0.596 (9)
C14	−0.0112 (19)	0.3748 (6)	0.5082 (14)	0.0496 (16)	0.596 (9)
H14	−0.0937	0.3561	0.4638	0.060*	0.596 (9)
C15	−0.067 (4)	0.3481 (8)	0.5740 (11)	0.0416 (17)	0.596 (9)
H15	−0.1855	0.3109	0.5753	0.050*	0.596 (9)
C16	0.0531 (17)	0.3765 (6)	0.6387 (12)	0.0348 (18)	0.596 (9)
H16	0.0109	0.3597	0.6843	0.042*	0.596 (9)
C12A	0.358 (2)	0.4353 (7)	0.5772 (6)	0.0463 (17)	0.404 (9)
H12A	0.5096	0.4589	0.5808	0.056*	0.404 (9)
C13A	0.253 (2)	0.4061 (6)	0.5123 (5)	0.0523 (15)	0.404 (9)
H13A	0.3295	0.4115	0.4695	0.063*	0.404 (9)
C14A	0.036 (3)	0.3687 (9)	0.508 (2)	0.0496 (16)	0.404 (9)
H14A	−0.0360	0.3486	0.4627	0.060*	0.404 (9)
C15A	−0.072 (6)	0.3615 (12)	0.5701 (17)	0.0416 (17)	0.404 (9)
H15A	−0.2206	0.3359	0.5668	0.050*	0.404 (9)
C16A	0.023 (3)	0.3894 (10)	0.6378 (19)	0.0348 (18)	0.404 (9)
H16A	−0.0513	0.3819	0.6808	0.042*	0.404 (9)
C17	0.9179 (4)	0.34677 (11)	0.84955 (11)	0.0281 (4)	
C18	1.1076 (17)	0.3082 (6)	0.8292 (8)	0.0342 (13)	0.787 (3)
H18	1.2341	0.3338	0.8103	0.041*	0.787 (3)
C19	1.1145 (8)	0.2330 (3)	0.8361 (3)	0.0375 (9)	0.787 (3)
H19	1.2400	0.2018	0.8242	0.045*	0.787 (3)
C20	0.9166 (7)	0.21043 (19)	0.8622 (3)	0.0430 (9)	0.787 (3)
H20	0.8838	0.1600	0.8722	0.052*	0.787 (3)
C18A	0.781 (3)	0.2960 (8)	0.8922 (9)	0.0385 (3)	0.213 (3)
H18A	0.6662	0.3094	0.9235	0.046*	0.213 (3)
C19A	0.853 (3)	0.2263 (9)	0.8774 (11)	0.0430 (9)	0.213 (3)
H19A	0.7666	0.1827	0.8860	0.052*	0.213 (3)
C20A	1.060 (4)	0.2264 (10)	0.8495 (14)	0.0375 (9)	0.213 (3)
H20A	1.1489	0.1825	0.8436	0.045*	0.213 (3)
C21	1.2205 (4)	0.44413 (11)	0.94061 (11)	0.0286 (4)	
N21	1.3785 (3)	0.42360 (10)	0.98069 (11)	0.0342 (4)	

### Atomic displacement parameters (Å^2^)

**Table:**

	*U*^11^	*U*^22^	*U*^33^	*U*^12^	*U*^13^	*U*^23^
S1	0.0299 (3)	0.0326 (3)	0.0293 (3)	0.00388 (19)	−0.00271 (19)	0.00214 (18)
S2	0.0288 (5)	0.0342 (5)	0.0513 (5)	−0.0065 (3)	−0.0005 (3)	0.0115 (3)
S2A	0.017 (3)	0.0302 (16)	0.0576 (16)	−0.0004 (19)	0.015 (2)	0.0102 (12)
O1	0.0358 (8)	0.0268 (7)	0.0350 (8)	0.0007 (6)	−0.0007 (6)	−0.0018 (6)
N1	0.0289 (8)	0.0287 (8)	0.0225 (8)	0.0041 (6)	−0.0015 (6)	0.0002 (6)
C1A	0.0269 (9)	0.0275 (10)	0.0214 (8)	0.0021 (7)	0.0015 (7)	0.0025 (7)
C2	0.0296 (9)	0.0283 (10)	0.0228 (9)	0.0001 (8)	0.0025 (7)	0.0028 (7)
N3	0.0302 (8)	0.0286 (8)	0.0243 (8)	0.0005 (7)	−0.0007 (6)	−0.0004 (6)
C4	0.0265 (9)	0.0262 (9)	0.0258 (9)	−0.0008 (7)	0.0022 (7)	0.0031 (7)
C4A	0.0251 (9)	0.0260 (9)	0.0239 (9)	−0.0007 (7)	0.0013 (7)	0.0038 (7)
C5	0.0243 (9)	0.0266 (9)	0.0255 (9)	−0.0003 (7)	0.0024 (7)	0.0055 (7)
C6	0.0263 (9)	0.0263 (10)	0.0260 (9)	0.0016 (7)	−0.0005 (7)	0.0043 (7)
C6A	0.0276 (9)	0.0280 (10)	0.0227 (9)	0.0024 (8)	0.0004 (7)	0.0015 (7)
N7	0.0318 (9)	0.0271 (9)	0.0341 (9)	0.0027 (7)	−0.0078 (7)	−0.0011 (7)
C8	0.0392 (12)	0.0266 (11)	0.0440 (12)	0.0024 (9)	−0.0090 (10)	−0.0045 (9)
C9	0.0430 (13)	0.0240 (11)	0.0316 (12)	0.0028 (9)	−0.0031 (9)	0.0007 (9)
C9A	0.0430 (13)	0.0240 (11)	0.0316 (12)	0.0028 (9)	−0.0031 (9)	0.0007 (9)
C10	0.0396 (11)	0.0239 (10)	0.0314 (10)	0.0067 (8)	−0.0072 (8)	−0.0012 (8)
N11	0.0300 (8)	0.0240 (8)	0.0233 (8)	0.0042 (6)	−0.0024 (6)	0.0001 (6)
C11	0.0369 (11)	0.0320 (11)	0.0287 (10)	−0.0004 (8)	0.0018 (9)	−0.0019 (8)
C12	0.051 (4)	0.054 (4)	0.0352 (18)	−0.016 (3)	0.008 (2)	−0.004 (2)
C13	0.061 (4)	0.067 (4)	0.0287 (17)	−0.010 (3)	0.003 (3)	−0.001 (2)
C14	0.056 (4)	0.053 (2)	0.0363 (13)	−0.010 (2)	−0.005 (4)	−0.007 (2)
C15	0.0428 (16)	0.034 (5)	0.045 (2)	−0.007 (4)	−0.0086 (15)	0.004 (3)
C16	0.031 (3)	0.037 (4)	0.0352 (13)	0.003 (3)	−0.001 (3)	0.004 (4)
C12A	0.051 (4)	0.054 (4)	0.0352 (18)	−0.016 (3)	0.008 (2)	−0.004 (2)
C13A	0.061 (4)	0.067 (4)	0.0287 (17)	−0.010 (3)	0.003 (3)	−0.001 (2)
C14A	0.056 (4)	0.053 (2)	0.0363 (13)	−0.010 (2)	−0.005 (4)	−0.007 (2)
C15A	0.0428 (16)	0.034 (5)	0.045 (2)	−0.007 (4)	−0.0086 (15)	0.004 (3)
C16A	0.031 (3)	0.037 (4)	0.0352 (13)	0.003 (3)	−0.001 (3)	0.004 (4)
C17	0.0271 (9)	0.0238 (9)	0.0315 (10)	−0.0025 (7)	−0.0044 (8)	0.0056 (8)
C18	0.017 (3)	0.0302 (16)	0.0576 (16)	−0.0004 (19)	0.015 (2)	0.0102 (12)
C19	0.042 (3)	0.0268 (14)	0.042 (3)	0.0073 (15)	−0.0043 (15)	−0.0022 (13)
C20	0.043 (3)	0.0223 (18)	0.059 (2)	−0.0055 (14)	−0.0132 (18)	0.0046 (15)
C18A	0.0288 (5)	0.0342 (5)	0.0513 (5)	−0.0065 (3)	−0.0005 (3)	0.0115 (3)
C19A	0.043 (3)	0.0223 (18)	0.059 (2)	−0.0055 (14)	−0.0132 (18)	0.0046 (15)
C20A	0.042 (3)	0.0268 (14)	0.042 (3)	0.0073 (15)	−0.0043 (15)	−0.0022 (13)
C21	0.0295 (10)	0.0248 (9)	0.0305 (10)	−0.0006 (8)	−0.0007 (8)	0.0024 (8)
N21	0.0330 (9)	0.0297 (9)	0.0369 (10)	0.0020 (7)	−0.0076 (8)	0.0026 (7)

### Geometric parameters (Å, º)

**Table:**

S1—C2	1.670 (2)	C10—H10C	0.9900
S2—C17	1.693 (2)	C10—H10D	0.9900
S2—C20	1.723 (4)	C11—C16A	1.377 (13)
S2A—C17	1.572 (9)	C11—C16	1.382 (9)
S2A—C20A	1.750 (17)	C11—C12	1.393 (7)
O1—C4	1.221 (2)	C11—C12A	1.400 (10)
N1—C1A	1.318 (3)	C12—C13	1.377 (7)
N1—C2	1.365 (3)	C12—H12	0.9500
C1A—N11	1.392 (3)	C13—C14	1.395 (9)
C1A—C4A	1.416 (3)	C13—H13	0.9500
C2—N3	1.397 (3)	C14—C15	1.374 (9)
N3—C4	1.398 (3)	C14—H14	0.9500
N3—C11	1.453 (3)	C15—C16	1.391 (9)
C4—C4A	1.464 (3)	C15—H15	0.9500
C4A—C5	1.397 (3)	C16—H16	0.9500
C5—C6	1.400 (3)	C12A—C13A	1.370 (11)
C5—C17	1.475 (3)	C12A—H12A	0.9500
C6—C6A	1.428 (3)	C13A—C14A	1.389 (13)
C6—C21	1.431 (3)	C13A—H13A	0.9500
C6A—N7	1.327 (3)	C14A—C15A	1.369 (14)
C6A—N11	1.360 (2)	C14A—H14A	0.9500
N7—C8	1.461 (3)	C15A—C16A	1.386 (13)
N7—H7	0.888 (9)	C15A—H15A	0.9500
C8—C9A	1.468 (16)	C16A—H16A	0.9500
C8—C9	1.496 (3)	C17—C18	1.366 (10)
C8—H8A	0.9900	C17—C18A	1.483 (14)
C8—H8B	0.9900	C18—C19	1.361 (11)
C8—H8C	0.9900	C18—H18	0.9500
C8—H8D	0.9900	C19—C20	1.332 (5)
C9—C10	1.517 (3)	C19—H19	0.9500
C9—H9A	0.9900	C20—H20	0.9500
C9—H9B	0.9900	C18A—C19A	1.359 (17)
C9A—C10	1.487 (16)	C18A—H18A	0.9500
C9A—H9C	0.9900	C19A—C20A	1.329 (16)
C9A—H9D	0.9900	C19A—H19A	0.9500
C10—N11	1.493 (3)	C20A—H20A	0.9500
C10—H10A	0.9900	C21—N21	1.145 (3)
C10—H10B	0.9900		
			
C17—S2—C20	92.57 (16)	H10C—C10—H10D	107.3
C17—S2A—C20A	88.1 (8)	C6A—N11—C1A	121.72 (17)
C1A—N1—C2	118.67 (17)	C6A—N11—C10	120.07 (17)
N1—C1A—N11	115.60 (17)	C1A—N11—C10	117.89 (16)
N1—C1A—C4A	124.97 (18)	C16—C11—C12	118.6 (10)
N11—C1A—C4A	119.43 (17)	C16A—C11—C12A	124.1 (15)
N1—C2—N3	119.04 (17)	C16A—C11—N3	120.5 (14)
N1—C2—S1	120.70 (15)	C16—C11—N3	121.3 (9)
N3—C2—S1	120.25 (15)	C12—C11—N3	119.9 (4)
C2—N3—C4	123.61 (17)	C12A—C11—N3	115.1 (5)
C2—N3—C11	119.51 (17)	C13—C12—C11	121.0 (6)
C4—N3—C11	116.66 (17)	C13—C12—H12	119.5
O1—C4—N3	119.91 (18)	C11—C12—H12	119.5
O1—C4—C4A	125.36 (18)	C12—C13—C14	119.2 (12)
N3—C4—C4A	114.69 (17)	C12—C13—H13	120.4
C5—C4A—C1A	120.10 (18)	C14—C13—H13	120.4
C5—C4A—C4	123.05 (18)	C15—C14—C13	121 (2)
C1A—C4A—C4	116.12 (17)	C15—C14—H14	119.6
C4A—C5—C6	118.50 (18)	C13—C14—H14	119.6
C4A—C5—C17	123.21 (18)	C14—C15—C16	119 (2)
C6—C5—C17	118.29 (17)	C14—C15—H15	120.5
C5—C6—C6A	121.15 (18)	C16—C15—H15	120.5
C5—C6—C21	121.27 (18)	C11—C16—C15	121.3 (17)
C6A—C6—C21	117.58 (18)	C11—C16—H16	119.3
N7—C6A—N11	120.44 (18)	C15—C16—H16	119.3
N7—C6A—C6	120.91 (18)	C13A—C12A—C11	117.1 (10)
N11—C6A—C6	118.63 (18)	C13A—C12A—H12A	121.4
C6A—N7—C8	124.19 (18)	C11—C12A—H12A	121.4
C6A—N7—H7	119 (2)	C12A—C13A—C14A	121.3 (19)
C8—N7—H7	116 (2)	C12A—C13A—H13A	119.3
N7—C8—C9A	115.6 (8)	C14A—C13A—H13A	119.3
N7—C8—C9	107.80 (18)	C15A—C14A—C13A	118 (3)
N7—C8—H8A	110.1	C15A—C14A—H14A	120.8
C9—C8—H8A	110.1	C13A—C14A—H14A	120.8
N7—C8—H8B	110.1	C14A—C15A—C16A	124 (3)
C9—C8—H8B	110.1	C14A—C15A—H15A	118.2
H8A—C8—H8B	108.5	C16A—C15A—H15A	118.2
N7—C8—H8C	108.4	C11—C16A—C15A	115 (3)
C9A—C8—H8C	108.4	C11—C16A—H16A	122.5
N7—C8—H8D	108.4	C15A—C16A—H16A	122.5
C9A—C8—H8D	108.4	C18—C17—C5	129.6 (5)
H8C—C8—H8D	107.4	C5—C17—C18A	121.3 (6)
C8—C9—C10	109.5 (2)	C5—C17—S2A	120.9 (4)
C8—C9—H9A	109.8	C18A—C17—S2A	116.0 (7)
C10—C9—H9A	109.8	C18—C17—S2	106.2 (5)
C8—C9—H9B	109.8	C5—C17—S2	124.18 (16)
C10—C9—H9B	109.8	C19—C18—C17	119.7 (7)
H9A—C9—H9B	108.2	C19—C18—H18	120.2
C8—C9A—C10	112.8 (12)	C17—C18—H18	120.2
C8—C9A—H9C	109.0	C20—C19—C18	108.8 (5)
C10—C9A—H9C	109.0	C20—C19—H19	125.6
C8—C9A—H9D	109.0	C18—C19—H19	125.6
C10—C9A—H9D	109.0	C19—C20—S2	112.7 (3)
H9C—C9A—H9D	107.8	C19—C20—H20	123.7
C9A—C10—N11	116.4 (8)	S2—C20—H20	123.7
N11—C10—C9	109.50 (17)	C19A—C18A—C17	106.0 (13)
N11—C10—H10A	109.8	C19A—C18A—H18A	127.0
C9—C10—H10A	109.8	C17—C18A—H18A	127.0
N11—C10—H10B	109.8	C20A—C19A—C18A	112.1 (16)
C9—C10—H10B	109.8	C20A—C19A—H19A	124.0
H10A—C10—H10B	108.2	C18A—C19A—H19A	124.0
C9A—C10—H10C	108.2	C19A—C20A—S2A	114.2 (15)
N11—C10—H10C	108.2	C19A—C20A—H20A	122.9
C9A—C10—H10D	108.2	S2A—C20A—H20A	122.9
N11—C10—H10D	108.2	N21—C21—C6	177.0 (2)
			
C2—N1—C1A—N11	172.45 (17)	C4—N3—C11—C16	83.5 (6)
C2—N1—C1A—C4A	−7.8 (3)	C2—N3—C11—C12	73.4 (5)
C1A—N1—C2—N3	−8.3 (3)	C4—N3—C11—C12	−101.4 (4)
C1A—N1—C2—S1	172.92 (15)	C2—N3—C11—C12A	98.0 (6)
N1—C2—N3—C4	14.5 (3)	C4—N3—C11—C12A	−76.8 (6)
S1—C2—N3—C4	−166.79 (15)	C16A—C11—C12—C13	−14.0 (12)
N1—C2—N3—C11	−159.97 (18)	C16—C11—C12—C13	−0.6 (9)
S1—C2—N3—C11	18.8 (3)	C12A—C11—C12—C13	101 (2)
C2—N3—C4—O1	177.90 (19)	N3—C11—C12—C13	−175.8 (4)
C11—N3—C4—O1	−7.5 (3)	C11—C12—C13—C14	−0.9 (10)
C2—N3—C4—C4A	−4.2 (3)	C12—C13—C14—C15	0.8 (11)
C11—N3—C4—C4A	170.37 (17)	C13—C14—C15—C16	0.8 (10)
N1—C1A—C4A—C5	−171.90 (19)	C16A—C11—C16—C15	87 (11)
N11—C1A—C4A—C5	7.8 (3)	C12—C11—C16—C15	2.3 (10)
N1—C1A—C4A—C4	17.6 (3)	C12A—C11—C16—C15	−23.3 (9)
N11—C1A—C4A—C4	−162.66 (17)	N3—C11—C16—C15	177.4 (6)
O1—C4—C4A—C5	−3.1 (3)	C14—C15—C16—C11	−2.4 (11)
N3—C4—C4A—C5	179.13 (18)	C16A—C11—C12A—C13A	6.1 (16)
O1—C4—C4A—C1A	167.1 (2)	C16—C11—C12A—C13A	19.7 (10)
N3—C4—C4A—C1A	−10.7 (3)	C12—C11—C12A—C13A	−71.8 (19)
C1A—C4A—C5—C6	−4.3 (3)	N3—C11—C12A—C13A	−179.9 (6)
C4—C4A—C5—C6	165.48 (18)	C11—C12A—C13A—C14A	−2.8 (7)
C1A—C4A—C5—C17	174.88 (18)	C12A—C13A—C14A—C15A	0.01 (9)
C4—C4A—C5—C17	−15.3 (3)	C13A—C14A—C15A—C16A	0.0 (2)
C4A—C5—C6—C6A	−2.0 (3)	C16—C11—C16A—C15A	−83 (11)
C17—C5—C6—C6A	178.80 (18)	C12—C11—C16A—C15A	18.6 (12)
C4A—C5—C6—C21	177.59 (19)	C12A—C11—C16A—C15A	−6.0 (16)
C17—C5—C6—C21	−1.7 (3)	N3—C11—C16A—C15A	−179.7 (5)
C5—C6—C6A—N7	−177.14 (19)	C14A—C15A—C16A—C11	2.9 (8)
C21—C6—C6A—N7	3.3 (3)	C4A—C5—C17—C18	115.8 (8)
C5—C6—C6A—N11	4.8 (3)	C6—C5—C17—C18	−65.0 (8)
C21—C6—C6A—N11	−174.77 (18)	C4A—C5—C17—C18A	−78.7 (8)
N11—C6A—N7—C8	−6.0 (3)	C6—C5—C17—C18A	100.5 (8)
C6—C6A—N7—C8	175.9 (2)	C4A—C5—C17—S2A	117.2 (6)
C6A—N7—C8—C9A	20.3 (11)	C6—C5—C17—S2A	−63.6 (6)
C6A—N7—C8—C9	−26.7 (3)	C4A—C5—C17—S2	−61.0 (3)
N7—C8—C9—C10	56.3 (3)	C6—C5—C17—S2	118.17 (19)
C9A—C8—C9—C10	−52.5 (11)	C20A—S2A—C17—C18	−10 (7)
N7—C8—C9A—C10	−34 (2)	C20A—S2A—C17—C5	177.4 (9)
C9—C8—C9A—C10	55.8 (12)	C20A—S2A—C17—C18A	12.5 (13)
C8—C9A—C10—N11	36 (2)	C20A—S2A—C17—S2	−4.3 (11)
C8—C9A—C10—C9	−55.6 (12)	C20—S2—C17—C18	2.6 (7)
C8—C9—C10—C9A	52.4 (11)	C20—S2—C17—C5	180.0 (2)
C8—C9—C10—N11	−55.8 (2)	C20—S2—C17—C18A	−96 (2)
N7—C6A—N11—C1A	−179.30 (19)	C20—S2—C17—S2A	1.7 (6)
C6—C6A—N11—C1A	−1.2 (3)	C5—C17—C18—C19	−179.9 (6)
N7—C6A—N11—C10	7.3 (3)	C18A—C17—C18—C19	13.1 (15)
C6—C6A—N11—C10	−174.60 (18)	S2A—C17—C18—C19	172 (8)
N1—C1A—N11—C6A	174.77 (18)	S2—C17—C18—C19	−2.7 (13)
C4A—C1A—N11—C6A	−5.0 (3)	C17—C18—C19—C20	1.2 (14)
N1—C1A—N11—C10	−11.7 (3)	C18—C19—C20—S2	1.0 (9)
C4A—C1A—N11—C10	168.53 (18)	C17—S2—C20—C19	−2.2 (4)
C9A—C10—N11—C6A	−23.0 (11)	C18—C17—C18A—C19A	−17.0 (16)
C9—C10—N11—C6A	23.9 (3)	C5—C17—C18A—C19A	174.7 (10)
C9A—C10—N11—C1A	163.4 (11)	S2A—C17—C18A—C19A	−20.5 (16)
C9—C10—N11—C1A	−149.71 (18)	S2—C17—C18A—C19A	69 (2)
C2—N3—C11—C16A	−87.8 (10)	C17—C18A—C19A—C20A	18 (2)
C4—N3—C11—C16A	97.4 (10)	C18A—C19A—C20A—S2A	−11 (3)
C2—N3—C11—C16	−101.7 (6)	C17—S2A—C20A—C19A	−1 (2)

### Hydrogen-bond geometry (Å, º)

**Table:**

*D*—H···*A*	*D*—H	H···*A*	*D*···*A*	*D*—H···*A*
N7—H7···N21^i^	0.89 (2)	2.14 (2)	2.976 (3)	157 (2)
C9—H9*B*···O1^ii^	0.99	2.34	3.197 (3)	144
C16—H16···S2*A*^iii^	0.95	2.73	3.58 (2)	149
C19—H19···S1^iv^	0.95	2.76	3.652 (5)	156

Symmetry codes: (i) −*x*+3, −*y*+1, −*z*+2; (ii) −*x*+3/2, *y*+1/2, −*z*+3/2; (iii) *x*−1, *y*, *z*; (iv) −*x*+3/2, *y*−1/2, −*z*+3/2.
